# Non-invasive diagnosis of vulvar dysplasia using cervical methylation markers—a case control study

**DOI:** 10.1186/s12916-025-03954-x

**Published:** 2025-02-28

**Authors:** Sabeth Becker, Lena Dübbel, Dana Behrens, Kristin Knoll, Juliane Hippe, Karin Loser, Eduard Malik, Meike Schild-Suhren

**Affiliations:** 1 University Clinic of Gynaecology and Obstetrics, Carl von Ossietzky Universität Oldenburg, Ammerländer Heerstraße 114-118, 26129 Oldenburg, Germany; 2 University Clinic of Gynaecology and Obstetrics, Klinikum Oldenburg, Rahel-Straus-Straße 10, 26133 Oldenburg, Germany; 3 oncgnostics GmbH, Löbstedter Str. 41, 07749 Jena, Germany; 4 Institute of Immunology, Carl von Ossietzky Universität Oldenburg, Ammerländer Heerstraße 114-118, 26129 Oldenburg, Germany

**Keywords:** Vulvar dysplasia, DNA methylation, GynTect®, Cervical dysplasia, Cancer of cervix and vulva, Epigenetic, Cervical smear, Vulvar smear

## Abstract

**Background:**

Diagnostic screenings for vulvar squamous intraepithelial lesions (VSIL) are limited and without information on disease trends. A panel of six methylation markers (ASTN1, DLX1, ITGA4, RXFP3, SOX17, ZNF671; GynTect® assay) has shown promise in diagnosing cervical intraepithelial neoplasia (CIN). Given the similarities between the carcinogenesis of cervix and vulva, this study aimed to investigate the suitability of these markers for diagnosing vulvar lesions.

**Methods:**

One hundred twenty-one vulvar FFPE samples and 237 vulvar cell smears with different VSIL grades, HPV status, and with or without lichen sclerosus and planus were tested. Additionally, dysplasia-free vulvar cell smears from patients with cervical dysplasia were analyzed. The expression of DNA methyltransferases (DNMTs) in the FFPE samples was measured.

**Results:**

The markers demonstrated high specificity in vulvar smears, with sole 5.45% of dysplasia-free smears testing positive. Yet, 75.00% of vulvar carcinoma smears appear positive in the methylation kit, similar to VHSIL (VIN III) smears with 77.78%. In FFPE samples, dysplasia-free samples from the tumor microenvironment of high-grade vulvar neoplasia showed 43.75% positivity. The positivity rates for VSIL and carcinoma samples were 76.92%, 64.71%, 64.71%, and 80.49%, respectively. DNMT3a expression was the highest in VLSIL (VIN I) samples, while DNMT1 was only expressed in VHSIL (VIN III) and carcinoma samples. Lichen sclerosis and planus showed a high false positive rate of 45.45% for dysplasia-free and 54.54% for smears with dVIN. Cervical HSIL was associated with a significantly higher number of positive results in the kit than in patients without cervical dysplasia.

**Conclusions:**

The findings suggest that the methylation markers comprising GynTect® may be suitable for detecting vulvar neoplasia, as they exhibit high sensitivity. Nonetheless, adjustments are needed for comparable specificity. Lichen should be considered in result interpretation, and the kit should be used with caution for patients with lichen. Moreover, we observed methylation changes as an early event with the highest positivity of VLSIL. Surprisingly, changes in methylation pattern are not as local as presumed. Cervical SIL led to changed methylation in the vulva. Patients with positive kit results should be monitored regularly for all genital dysplasia. This sheds new light on the epigenetics in cancer.

**Supplementary Information:**

The online version contains supplementary material available at 10.1186/s12916-025-03954-x.

## Background

Vulvar squamous cell carcinoma (VSCC) is generally considered a rare cancer. However, in recent decades its incidence increased and the median age of onset decreased [1–5]. The precursor lesion to VSCC is the vulvar squamous intraepithelial lesion (VSIL), which can be further subdivided into low-grade SIL (VLSIL, formerly vulvar intraepithelial neoplasia (VIN) I) and high-grade SIL (VHSIL, formerly VIN II and III). VSIL is related to human papillomavirus (HPV). Differentiated VIN (dVIN) is independent of HPV and associated with lichen sclerosus and lichen planus [6–8]. HSIL (VIN II and III) is the most common type of VIN, primarily affecting women aged between 35 and 50 years. Despite the relatively low absolute cancer risk of VHSIL, ranging between 2.3 and 6.6% after 3 years, all VHSIL (VIN II and III) are treated to prevent cancer [9–11]. VLSIL (VIN I), a synonym for condyloma plana, is caused by both high- and low-risk HPV. It has a high spontaneous remission rate and is a precancerous lesion with only a low risk of VSCC [6]. In contrast, dVIN has a higher oncogenic potential, which can be as high as 43.2% even after treatment [12]. A differentiation according to the earlier classification VIN I, II, and III allows a better discrimination of the dysplasia grades. It is therefore used for this study additionally to the standard classification to further discriminate the VHSIL section into VHSIL (VIN II) and VHSIL (VIN III). VHSIL is treated in order to exclude foci of invasion and lower the risk of progression to cancer [13–15]. Unfortunately, surgical interventions in the vulva can damage adjacent vital structures, leading to post-operative morbidity and a reduced quality of life [16, 17]. To reduce overtreatment and associated morbidity, biomarkers that could predict individual cancer risk in women with VHSIL are urgently needed. Epigenetic changes, such as hypermethylation of promoter cytosine-phosphate-guanine (CpG) islands of tumor suppressor genes, can contribute to the development of cancer by gene silencing [18]. In recent years, several studies have investigated genomic changes in VSCC and VSIL [19–24]. However, the epigenomic changes in VSCC remain relatively underexplored. Deoxyribonucleic acid (DNA) methylation testing has provided promising biomarkers in HPV-related cervical and anal diseases for identifying precursors with a presumed high cancer risk [18, 25]. Various methylation markers associated with HPV-induced anogenital carcinogenesis have been discovered, including *ASTN1*, *DLX1*, *ITGA4*, *RXFP3*, *SOX17*, and *ZNF671* [26]. Currently, GynTect® by oncgnostics GmbH and the QIAsure Methylation Test are the two commercially available methylation tests for cervical dysplasia, with GynTect® showing the higher specificity [27]. Such diagnostic and prognostic aids are not yet available for vulvar dysplasia. DNA methyltransferases (DNMTs) are responsible for DNA methylation [28]. HPV influences DNMT expression mainly via E6 and E7 and thus potentially promotes carcinogenesis [29].

This study aimed to apply a panel of six methylation markers to vulvar FFPE tissue and to explore the potential for non-invasive vulvar dysplasia diagnostics using vulvar smears.

## Methods

Six methylation markers were assessed using the GynTect® kit, to transfer the markers with very high sensitivity and highest specificity from cervical to vulvar samples. Both a calculation in which all dysplasias were evaluated as positive and a calculation in which only the high-grade dysplasias were evaluated as positive were carried out. To clarify pathogenesis, an expression analysis of DNA methyltransferases was performed. Samples of lichenoid vulvar disease with and without dVIN were tested. The HPV status was correlated with all results. Vulvar and cervical samples from patients with cervical intraepithelial neoplasia (CIN) were tested with the methylation kit and the results were compared.

### Ethics statements

All patients were diagnosed and managed in the Department of Gynaecology and Obstetrics at Klinikum Oldenburg University Hospital, and they agreed in written form to use the retrieved samples and data for research purposes. This study was approved by the Medical Ethics Committee of the Carl-von-Ossietzky University of Oldenburg (Ethical vote no. 2017-114 and 2020-187) and complies with the ethical principles for medical research of the Declaration of Helsinki. This retrospective study is registered at the German Clinical Trials Register (DRKS00024987).

### Study population and sample collection

All samples were obtained from patients visiting the dysplasia unit or emergency department in the Department of Gynaecology and Obstetrics at Klinikum Oldenburg University Hospital. For this study, formalin-fixed paraffin-embedded tissue and vulvar smears diluted in 10 ml cell collection medium (Roche Diagnostics, Basel, Switzerland) were used. Moreover, seven fresh frozen vulvar carcinoma samples obtained during surgery were studied. Histological diagnosis was performed by the Institute of Pathology Oldenburg.

For all patients, age, biological group (control, control with cervical dysplasia, VLSIL (VIN I), VHSIL (VIN II, VIN III), VSCC), lichen status (lichen sclerosus, lichen planus, with/without dVIN), and HPV status (HPV-positive, HPV-unknown, HPV-negative) were evaluated. Pregnant patients were excluded from this study.

In total, 121 FFPE samples, 237 fresh vulvar cell smears, and seven samples of fresh vulvar carcinoma were used in this study.

FFPE: The tissue, initially obtained by biopsy or excision, was fixed in formalin and embedded in paraffin by the Institute of Pathology Oldenburg for diagnostic analysis. After pathologists diagnosed the SIL grading (resp. VIN grading), the FFPE material was transported to the workgroup laboratory and stored at room temperature until analysis.

Vulvar smears: To obtain the samples, vulvar smears were taken during the dysplasia consultation or during the gynecological or emergency consultation. Various collection devices (brushes, spatulas, and cotton pads of various materials) were tested for vulvar smears before the start of the study. A conventional cervical brush made of polyethylene (Cervex-Brush® Combi, Rovers Medical Devices, Oss, Netherlands) was able to non-invasively generate an adequate number of vulvar cells per smear and was used for all patients subsequently. After taking the vulvar smear, the cell material was carefully transferred to 10 ml cell collection medium (Roche Diagnostics, Basel, Switzerland), and the brush was discarded. The cell material was stored at 4 °C and analyzed within 12 weeks.

Fresh frozen vulvar carcinoma: Seven samples of fresh vulvar carcinoma were collected during surgery from the punctum maximum of the vulvar carcinoma in liquid nitrogen. After transportation to the workgroup laboratory, the tissue has been stored at −150 °C until analysis.

### Methylation kit (GynTect®)

#### DNA isolation and bisulfite treatment

FFPE: Vulvar FFPE samples were cut in 10 µm thick sections. They were then processed with the EpiTect Fast FFPE Bisulfite Kit (Qiagen, 59844), which consists of the EpiTect Fast FFPE Lysis Kit for deparaffinization and lysis of FFPE tissue slices, and the EpiTect Fast DNA Bisulfite Kit for bisulfite conversion of the extracted DNA. Work was performed according to the manufacturer’s protocol.

Vulvar/cervical smears: First, the cells were concentrated in the medium: To stir the cervical cells up, the sample was vortexed for 5 s. Vulvar smears showed a much lower cell number compared to cervical smears; therefore, the concentrated cells from the bottom of the tube were used. Therefore, 1.5 ml of the sample was transferred into a 1.5 ml reaction tube and centrifuged at room temperature for 5 min at 10.000 ×g. The supernatant was removed and the procedure was repeated. The cells were resuspended in 40 µl of remaining supernatant and directly processed with the EpiTect Fast DNA Bisulfite Kit (Qiagen, Hilden, Germany) according to the manufacturer’s protocol for bisulfite conversion and cleanup of genomic DNA.

Fresh frozen vulvar carcinoma: The fresh frozen vulvar carcinoma tissue was first defrosted and homogenized, then the EpiTect Fast DNA Bisulfite Kit (Qiagen, Hilden, Germany) was performed according to the manufacturer’s protocol.

#### Methylation-specific PCR

The GynTect® real-time methylation-specific polymerase chain reaction (PCR) (qMSP) setup was performed for *ACHE*, *IDS-M*, *ASTN1*, *DLX1*, *ITGA4*, *RXFP3*, *SOX17*, and *ZNF671* according to the manufacturer’s instructions using the CFX Connect Real-Time PCR system (Bio-Rad Laboratories, Hercules, USA).

1. 94 °C for 1 min
2. 94 °C for 15 s
3. 66 °C for 35 s
4. Measurement of fluorescence
5. Repeat steps 2–4 41 times
6. 95 °C for 15 s
7. Melt curve 60–95 °C, increment 0.5 °C every 5 s

For the qRT-PCR, the real-time PCR program CFX MaestroTM (Bio-Rad) was used. Further analyses of the data were carried out in Microsoft Excel.

#### Scoring of the markers

After quantitative real-time (qRT)-PCR, the ∆ was calculated between the cycle threshold (Ct) value of the quality control markers *ACHE* and *IDS-M* and the Ct value for each of the six methylation markers. Each marker is assigned a score based on the probability of the marker being methylated only in precancerous or cancerous cervical tissue [26]. The scores of those markers with a ∆Ct within a certain range were summed up. The evaluation was carried out in accordance with the GynTect® Information for Users:

- GynTect® negative: score of the methylation marker genes ≤ 5
- GynTect® positive: score of the methylation marker genes ≥ 6
- GynTect® invalid: Ct value or melting temperature of one or both of the control markers *ACHE* and/or *IDS-M* out of range

We adapted one aspect of the original protocol due to the limited cell material of vulvar smears compared to cervical smears. All samples that showed positive marker results summing up to a positive score were considered positive, not invalid, even if the *IDS-M* Ct value was higher than 32 which is the maximum for validity normally.

#### Evaluation of test quality criteria

In addition to the overall test performance, the test quality criteria for each individual methylation marker were calculated. Sensitivity and specificity (Sens., Spec.) [30], positive and negative predictive value (PPV, NPV), prevalence, and positive and negative diagnostic likelihood ratio (DLR+, DLR−) [31] have been calculated. Both a calculation in which all dysplasia (VLSIL and VHSIL) were classified as positive as main analysis and a calculation with only the higher-grade dysplasia (VHSIL) classified as positive as additional analysis were carried out. Eleven samples with dVIN were deliberately not included in the analysis, as dVIN are not associated with hrHPV. However, based on the interim results, the decision was made to additionally analyze dVIN samples during the course of the study. Interim results already revealed that the methylation status seemed to be independent of the hrHPV status. Therefore, dysplasia not associated with hrHPV (dVIN) was additionally examined to test the theory that there seemed to be no hrHPV-associated methylation in vulvar tissue.

The following criteria were used for test quality criteria calculation:

- Main analysis:
-
  - Test negative: Methylation kit negative (GynTect® score ≤ 5); quality criteria of qRT-PCR not met and/or no expression of the marker in qRT-PCR
  - Test positive: Methylation kit positive (GynTect® score ≥ 6); Ct value within the limit of 20–42
  - Diagnosis positive: VIN I, II, III or VSCC
  - Diagnosis negative: Dysplasia-free sample
- Additional analysis:
-
  - Test negative: Methylation kit negative (GynTect® score ≤ 5); quality criteria of qRT-PCR not met and/or no expression of the marker in qRT-PCR
  - Test positive: Methylation kit positive (GynTect® score ≥ 6); Ct value within the limit of 20–42
  - Diagnosis positive: VHSIL (VIN II, III) or VSCC
  - Diagnosis negative: Dysplasia-free sample or VLSIL (VIN I)

### DNA methyltransferases

#### RNA isolation, DNase digestion, and cDNA synthesis

Sixteen-micrometer paraffin sections of FFPE samples were collected in a 1.5 ml reaction tube. For dewaxing, the samples were incubated 2–3 times for 5 min in xylene, followed by two times ethanol for 5 min. Next, diethyl pyrocarbonate treated (DEPC) H_2_O was added. Tissue was lysed with 750 µl Digestion Solution (100 g Guanidin-Thiocyanat + 6 ml 1 M Tris-HCl pH 7.6 + 13.3 ml 30% Na-N-Laury-Sarcosine), 300 µl Proteinase K solution (500 mg per 25 ml DEPC-H_2_O, Merck, 1245680100), and 5.5 µl β-mercaptoethanol (Merck, 1245680100). The tissue was incubated overnight at 1500 rpm and 55 °C. Six hundred thirty microliters of Roti® Aqua-Phenol (pH < 4, Carl Roth, A980.1), 270 µl chloroform, and 100 µl 3 M sodium acetate (pH 5.5) were added and incubated for 15 min on ice and afterwards centrifuged at 16,000 ×g for 20 min at 4 °C. RNA was transferred into new 2 ml reaction tubes and 1000 µl isopropanol and 1 µl glycogen were added and incubated for 1 h. The tubes were centrifuged at 16,000 ×g for 20 min at 4 °C and the pellets were washed twice by adding 1000 µl of 75% ethanol and centrifuging at 16,000 ×g for 15 min at 4 °C. The RNA pellet was resuspended in 10 µl DEPC-H_2_O.

The DNase digestion and cDNA digestion were performed with the iScript gDNA Clear cDNA Synthesis Kit (BioRad, 172-5035) according to the manufacturer’s manual.

#### qRT-PCR

To analyze the gene expression of DNMT1 and DNMT3a, qRT-PCRs were performed with the cDNA samples. As housekeeping genes, glyceraldehyde 3-phosphate dehydrogenase (*GAPDH*; annealing temp. 58 °C, primer concentration 300 nM, 75 ng cDNA) and ribosomal protein lateral stalk subunit P0 (*RPLP0*; annealing temp. 59.1 °C, primer concentration 250 nM, 75 ng cDNA) were used. For *DNMT1*, the annealing temperature was 58.7 °C, the primer concentration 450 nM, and the required amount of cDNA was 25 ng. For *DNMT3a*, the annealing temperature was 57.9 °C, the primer concentration 450 nM, and the required amount of cDNA was 75 ng. If possible, three technical replicates were made for each sample. For each plate, three technical replicates of one no-template control (NTC) and one no reverse transcriptase control (NRT) were performed.

qRT-PCR was performed on the CFX Connect Real-Time PCR system (Bio-Rad Laboratories, Inc., USA) applying the following PCR protocol:

1. 95 °C for 3 min
2. 95 °C for 5 s
3. Annealing temperature of the used primer set (see above) for 30 s
4. Measurement of fluorescence
5. Repeat steps 2–4 40 times
6. Melt curve 65–95 °C, increment 0.5 °C every 5 s

### Analysis and statistics

Due to uneven sample sizes, many results were analyzed in percentages. However, data in percentages is not normally distributed. Therefore, the chi-square test was used to test the stochastic independence of the two variables of the plots [31]. If the null hypothesis was true, the variables were considered stochastically independent. To test that, observed frequencies of the variables were created with sums of every column and row. The expected frequencies were calculated as *f* = (sum of the row * sum of the column) / sample size. Afterwards, the chi-square value was calculated with $\upchi$ ^2^ = (observed frequency − expected frequency)^2^ / expected frequency. The degrees of freedom were calculated with (number of columns − 1) * (number of rows − 1). Depending on the degrees of freedom and the significance of at least 0.95, the critical $\upchi$ ^2^ value was determined. If the calculated $\upchi$ ^2^ value was larger than the critical $\upchi$ ^2^ value, the two tested variables were considered to be significantly different.

qRT-PCR was performed and analyzed with the real-time PCR program CFX MaestroTM (BioRad). Gene studies in the program were used to display and evaluate the data. The statistical significance of the qRT-PCR results was tested with a one-way ANOVA. The *p* value threshold was set at 0.05, and the confidence interval was 95%. A Shapiro-Wilk normality test [32] was performed to ensure normal sample distribution. If normal distribution was confirmed, Tukey’s post hoc test was used to assess the significance of differences between mean gene expression values. CFX MaestroTM uses the 2^−∆∆Ct^ method to calculate the relative fold gene expression [33].

The scores of the seven fresh frozen carcinomas were compared with the FFPE samples from the same patient using a paired *t*-test.

## Results

### Patient population

One hundred twenty-one FFPE patient samples were included in this study:

- 16 of those were dysplasia-free
- 13 showed VLSIL (VIN I)
- 17 VHSIL (VIN II)
- 34 VHSIL (VIN III)
- 41 with VSCC diagnosis

We compared our results of FFPE VSCC to seven fresh frozen vulvar carcinoma samples to observe the impact of fixation on our results.

On top of that, we included 237 fresh vulvar cell smears of patients:

- 110 dysplasia-free vulvar smears, 9 VHSIL (VIN III) smears, and 12 VSCC smears
- 11 dysplasia-free vulvar smears with lichen (7 with lichen sclerosus, 4 with lichen planus) and 11 vulvar smears with dVIN and lichen (9 with lichen sclerosus, 2 with lichen planus)
- 84 dysplasia-free vulvar smears of patients with cervical dysplasia (11 with CLSIL (CIN I), 25 with CHSIL (CIN II), 45 with CHSIL (CIN III), and 3 with carcinoma/ACIS). For 54 of those patients we collected the cervical smears as well

The detailed patient information is included in Additional file 1: Table S1.

### Transfer of GynTect® to vulvar FFPE tissue

**Fig. 1:**
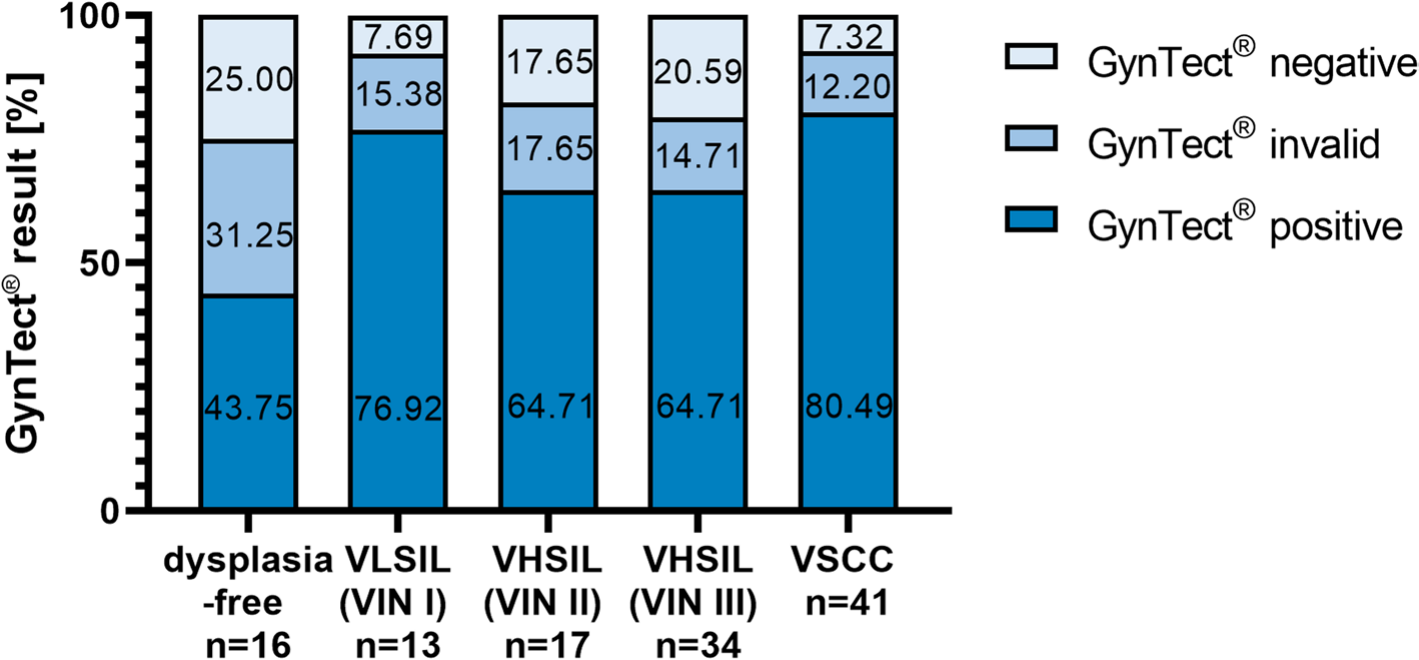
GynTect® performance on FFPE material. The variables differ significantly according chi-square test (*p* = 0). FFPE, formalin-fixed paraffin-embedded; VHSIL, vulvar high-grade squamous intraepithelial lesion; VIN, vulvar intraepithelial neoplasia; VLSIL, vulvar low-grade squamous intraepithelial lesion; VSCC, vulvar squamous cell carcinoma

Due to different sample numbers, percentages of GynTect® positive, negative, and invalid samples were calculated. 13.22% of the samples were dysplasia-free (*n* = 16) but isolated from the tumor microenvironment during surgeries of high-grade vulvar neoplasias. That is why they show a high positivity in GynTect® of 43.75% (*n* = 7). Another 31.25% (*n* = 5) were invalid and 25.00% (*n* = 4) negative in the GynTect® kit. 10.74% of the FFPE samples were VLSIL (VIN I) samples (*n* = 13) with 76.92% positive samples (*n* = 10), 15.38% invalid samples (*n* = 2), and 7.69% negative samples (*n* = 1). We had 14.05% VHSIL (VIN II) samples (*n* = 17), with 64.71% positive (*n* = 11), and 17.65% each invalid and negative samples (*n* = 3) samples in the GynTect® kit. 64.71% of all VHSIL (VIN III) samples (*n* = 27, 22.31% of samples) were positive and 14.71% were invalid (*n* = 5) and 20.59% were GynTect®-negative (*n* = 7). VSCC samples (*n* = 41, 33.88% of samples) showed 80.49% GynTect® positivity (*n* = 33), 12.20% invalid samples (*n* = 5), and 7.32% negative samples (*n* = 3) (Fig. 1). The variables differ significantly according chi-square test (*p* = 0) (Additional file 2: Table S2).

An overview of the average scores of the methylation panel across the biological groups is shown in Additional file 3: Fig. S2. Moreover, in order to enable an unbiased assessment of the methylation patterns in vulvar lesions independent of the kit thresholds and scores, an overview of the Ct values for the methylated markers from the panel across the biological groups is presented in Additional file 3: Fig. S3. Here we would like to refer to the single marker analysis in the supplements, which together with Additional file 3: Fig. S3 provides a good overview of the individual markers and their expression level and performance in the biological groups.

To prove that GynTect® results from FFPE samples are reliable and not influenced by the degenerated nucleic acids due to fixation, a total of 7 fresh frozen carcinoma tissues for which FFPE samples were tested already were examined. The scores of the methylation kit for the fresh frozen carcinoma were compared with those of the respective FFPE sample.

All seven fresh carcinomas were positive for GynTect® methylation. When comparing the scores of the fresh carcinomas with the scores of the corresponding FFPE blocks, the *t*-test showed no significant difference between the groups (*p* = 0.32). Sample 7 differs most in the kit result between fresh tissue and FFPE (score 9 in FFPE versus 15 in fresh frozen carcinoma). The scores are plotted against each other in Additional file 3: Fig. S1.

#### Correlation of GynTect® results and DNMT expression

**Fig. 2:**
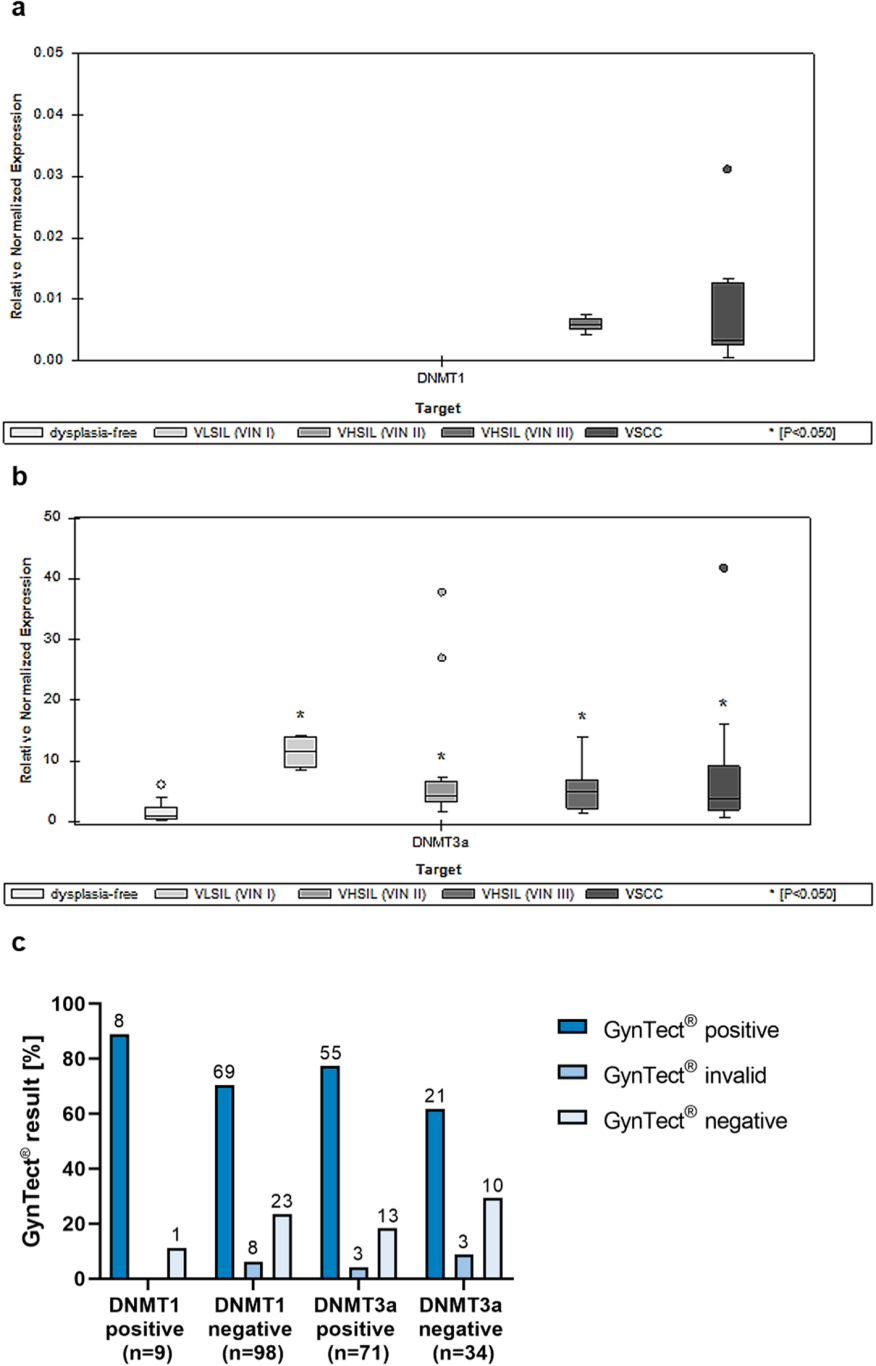
Boxplots of qRT-PCR expression of DNMT1 (**a**) and DNMT3a (**b**) in FFPE samples. Data is shown normalized to the housekeeping genes *GAPDH* and *RPLP0* and dysplasia-free tissue as a control. Median values, 95% interval ± SEM are shown. Correlation of DNMT expression and GynTect® results in **c**. Number *n* on bars. DNMT, DNA methyltransferase; FFPE, formalin-fixed paraffin-embedded; qRT-PCR, quantitative real-time polymerase chain reaction; VHSIL, vulvar high-grade squamous intraepithelial lesion; VIN, vulvar intraepithelial neoplasia; VLSIL, vulvar low-grade squamous intraepithelial lesion; VSCC, vulvar squamous cell carcinoma

We analyzed the expression of the DNA methyltransferases 1 and 3a in the FFPE samples via qRT-PCR. DNMT1 was only expressed in 9 of the 107 FFPE samples with successful qRT-PCR, which were all VHSIL (VIN III) and VSCC (Fig. 2a) with higher expression in VHSIL (VIN III). There was no expression of DNMT1 in the controls, VLSIL (VIN I), and VHSIL (VIN II) samples. Furthermore, there was no significant difference between the DNMT1 expression of VHSIL (VIN III) and VSCC samples (*p* = 0.98. 95%-width of confidence interval (CI): 0.10–14.13). Eight of 9 samples which showed DNMT1 expression were GynTect® positive (Fig. 2c).

DNMT3a was broadly expressed in 71 of the 105 FFPE samples with successful qRT-PCR in all biological groups. We saw significantly higher expression in all dysplasia grades compared to the dysplasia-free samples (each *p* < 0.001) (Fig. 2b). There was no significant difference between the dysplasia grades. The fold change of relative normalized DNMT3a expression was highest in VLSIL (VIN I) and decreased with increasing dysplasia severity. The *p* values were below 0.05 and therefore significant for all dysplasia grades. For details, see Table 1.

**Table 1:** qRT-PCR expression fold change and *p* value of DNMT3a in vulvar FFPE samples. *T*-test for significance for all dysplasia grades to dysplasia-free samples

Dysplasia grade	Fold change relative to dysplasia-free samples	*p* value
VLSIL (VIN I)	10.99	0.001
VHSIL (VIN II)	5.26	0.001
VHSIL (VIN III)	4.13	0.000
VSCC	3.88	0.000

Fifty-five of 71 FFPE samples which showed DNMT3a expression were GynTect®-positive, 11 showed negative GynTect® results, and 5 invalid GynTect® results (Fig. 2c).

#### Correlation of GynTect® results and HPV status

Of the total 121 FFPE samples, 43.80% (*n* = 53) were high-risk (hr) HPV-positive and 38.02% (*n* = 46) were hrHPV-negative. In 18.18% (*n* = 22), the HPV status was not known.

**Fig. 3:**
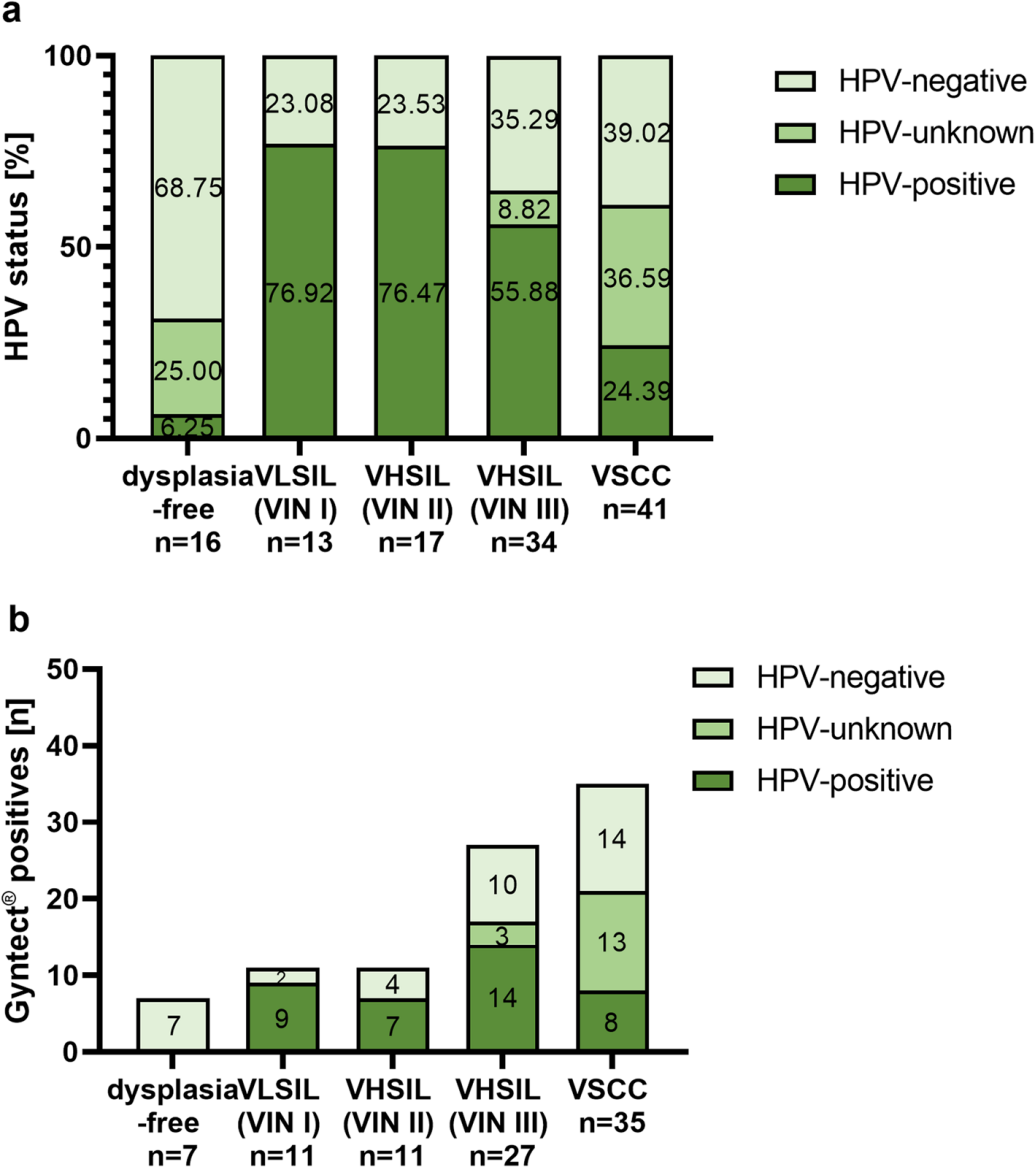
HPV status of FFPE vulvar samples (**a**). HPV status of FFPE samples with positive GynTect® result (**b**). HPV, human papillomavirus; FFPE, formalin-fixed paraffin-embedded; VHSIL, vulvar high-grade squamous intraepithelial lesion; VIN, vulvar intraepithelial neoplasia; VLSIL, vulvar low-grade squamous intraepithelial lesion; VSCC, vulvar squamous cell carcinoma

While only 6.25% (*n* = 1) of the dysplasia-free samples were HPV-positive, the proportion in VLSIL (VIN I) (*n* = 10) and VHSIL (VIN II) (*n* = 13) was highest with more than 76% each (Fig. 3a). The proportion of HPV-positive samples decreased again with rising dysplasia grade and carcinoma, as VHSIL (VIN III) samples were HPV-positive in 55.88% (*n* = 19), and VSCC in 24.39% (*n* = 10) of the cases. The higher the dysplasia grade, the lower the proportion of HPV-positive samples. When only the GynTect®-positive samples were analyzed, all 7 dysplasia-free GynTect®-positive were HPV-negative (Fig. 3b). Of the 11 VLSIL (VIN I) samples with positive kit results, 9 were HPV-positive and 2 HPV-negative. For the 11 VHSIL (VIN II) samples with GynTect®-positive results, 7 were HPV-positive and 4 HPV-negative. Among the 27 VHSIL (VIN III) samples that were positive for GynTect®, 14 showed an HPV-positive status, 10 an HPV-negative status, and for 3 samples, the HPV status was unknown. Of the 35 VSCCs that showed GynTect®-positive results, 8 were HPV-positive, 14 were HPV-negative, and 13 had unknown HPV status.

### Transfer of the GynTect® results to fresh vulvar smears

Due to the rare occurrence of vulvar dysplasia and the limited time of smear collection of 1 year, we could only collect smears from 9 VHSIL (VIN III) and 12 VSCC patients. In addition, we collected 110 dysplasia-free vulvar smears to assess whether the GynTect® kit is applicable with these samples and specifically recognizes dysplasia-free smears as negative.

**Fig. 4:**
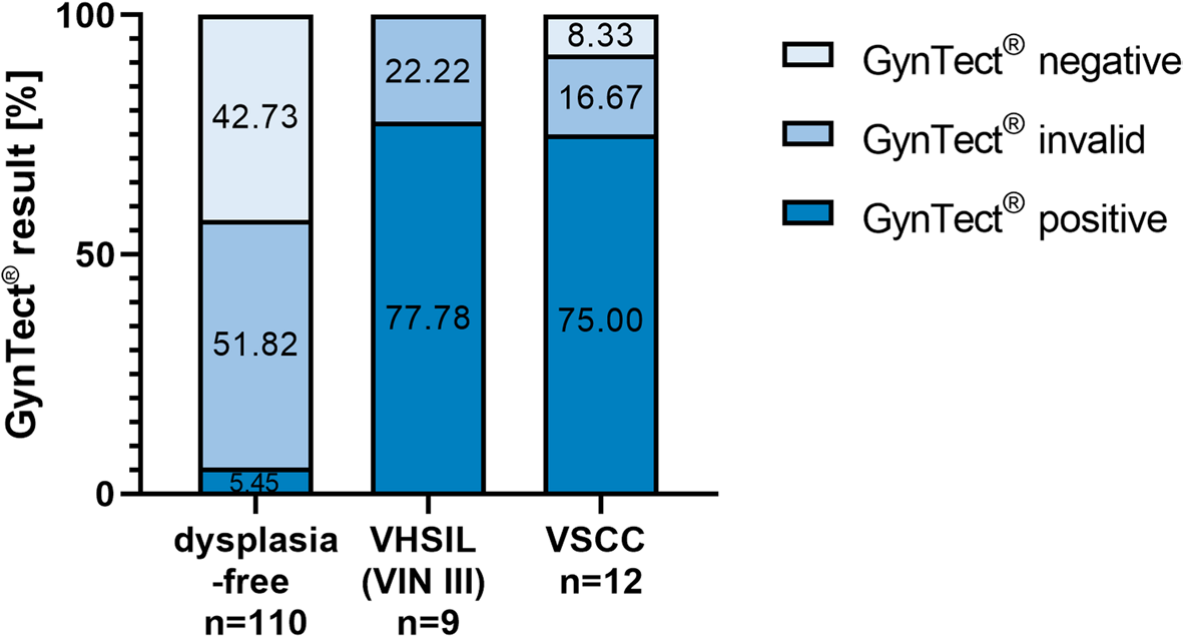
GynTect® performance on vulvar smears. The variables differ significantly according chi-square test (*p* = 0). VHSIL, vulvar high-grade squamous intraepithelial lesion; VIN, vulvar intraepithelial neoplasia; VSCC, vulvar squamous cell carcinoma

Of all 110 dysplasia-free samples, 47 were negative (42.73%), 57 invalid (51.82%), and 6 positive (5.45%) in the methylation kit (Fig. 4). 84.55% (*n* = 93) of the dysplasia-free samples were from patients in the emergency department or peripheral ward, and 15.45% (*n* = 17) from the dysplasia unit. Each of the 6 dysplasia-free samples that were positive in the methylation kit was taken at the dysplasia unit.

Most of the six positive patients had a risk constellation:

- Patient 1 had a vulvar carcinoma almost 2 years prior (11/2022) and a VHSIL (VIN III) (04/2023) 1 year prior and presented to rule out a recurrence. The histology was without dysplasia and the sample was assigned to the dysplasia-free samples in a blinded manner.
- The 2nd patient presented with suspected CIN II in cytology and HPV 18 persistence; the histology was unremarkable. The vulvar smear was therefore classified as dysplasia-free. She has a history of HPV-induced oral floor carcinoma.
- The third patient presented for a check-up after flat condylomas in 2019 and genital herpes (HSV 2) in 2023. Histology revealed only a discrete lichen simplex without dysplasia.
- The fourth patient also came for a check-up with suspected CIN II in cytology. She was post laser excision and vaporization (09/2022, 04/2023, and 08/2023) for CHSIL (CIN III) and HPV 16. The histology revealed no dysplasia.
- The fifth patient had a dysplasia-free acute ulceration on histology when the smear was taken but developed a VHSIL (VIN III) just 1 month after the smear.
- The last patient came for a check-up with a persistent hrHPV infection in the cervix. Cytology and colposcopy were unremarkable.

The 9 VHSIL (VIN III) samples showed 77.78% positivity (*n* = 7) and 22.22% invalid results (*n* = 2) in the GynTect® kit. VSCC smears showed a comparable positivity with 75.00% (*n* = 9), 16.67% invalid samples (*n* = 2), and 8.33% negative samples (*n* = 1) (Fig. 4). The variables differ significantly according to the chi-square test (*p* = 0) (Additional file 2: Table S2). Compared to cervical carcinoma, we did not see a 100% recognition of carcinoma smears in the GynTect® kit.

Looking at the performance of the individual markers (see Additional file 1 for more detailed information), the best-performing marker was *ASTN1*. In the kit, *ASTN1* was given a score of 2. *ZNF671*, which has the highest score of 6 points in the cervix GynTect® kit, performed in vulvar tissue not as good as in cervical tissue. *DLX1* is by far the worst-performing marker in vulvar tissue showing a positivity of 80% in dysplasia-free controls. It offers no additional diagnostic value and should therefore not be used in a vulvar methylation kit.

#### Smears of lichen sclerosus and planus with and without dVIN in the methylation kit

**Fig. 5:**
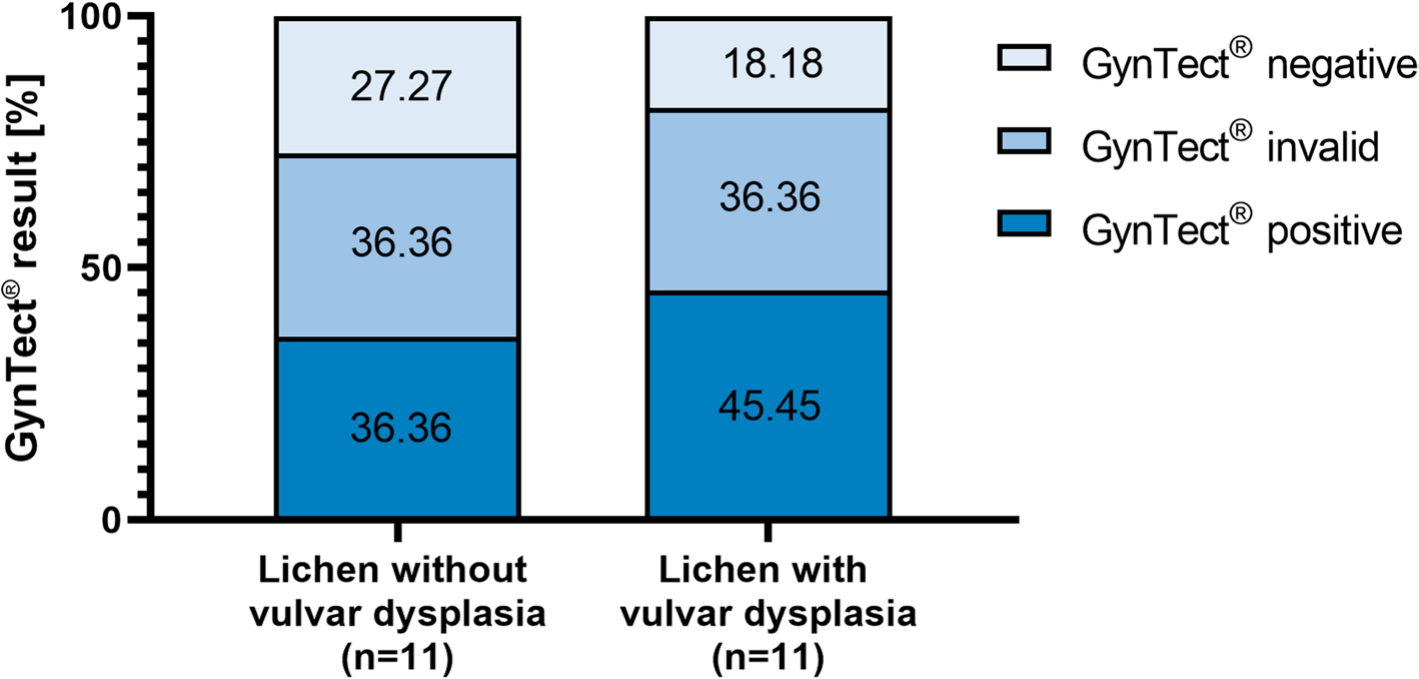
Vulvar smears from patients with lichen sclerosus or planus

We also investigated the positivity of vulvar smears with lichen sclerosus or lichen planus with and without dVIN (Fig. 5). Dysplasia-free lichen smears showed positivity of 36.36% (*n* = 4), invalid samples were 36.36% (*n* = 4), and negative samples were 27.27% (*n* = 3). A dVIN on top of lichen sclerosis or planus increased the positivity to 45.45% (*n* = 5), invalid samples were 36.36% (*n* = 4), and negative samples were only 18.18% (*n* = 2). In view of the fact that the panel ran with smears rather than histological sampling, we consider this to be quite acceptable. A slightly lower methylation level than average can be expected due to the keratosis and therefore a reduced amount of nuclei.

### Performance comparison of FFPE and vulvar smears in the GynTect® kit

In the main analysis, where all vulvar dysplasias were classified as “diagnosis positive” (VLSIL (VIN I) and VHSIL (VIN II and III)), the methylation kit showed a sensitivity of 94.44% (95%-width of CI: 87.51–98.17%) and a specificity of 40.0% (95%-width of CI: 12.16–73.76%) for FFPE tissue. However, with a positive likelihood ratio (DLR+) of 1.574 (95%-width of CI: 0.947–2.617) and a negative likelihood ratio (DLR−) of 0.139 (95%-width of CI: 0.044–0.435), test performance was rated poor to fair for DLR+ and good for DLR−.

In the additional analysis, only high-grade vulvar dysplasias were classified as “diagnosis positive” (VHSIL (VIN II and III)), whereas low-grade dysplasia (VLSIL) and dysplasia-free tissue were classified as “diagnosis negative.” The methylation kit showed a sensitivity of 97.37% (95%-width of CI: 90.82–99.68%) and a specificity of 29.17% (95%-width of CI: 12.62–51.09%) for FFPE tissue. A positive likelihood ratio (DLR+) of 1.375 (95%-width of CI: 1.081–1.782) and a negative likelihood ratio (DLR−) of 0.09 (95%-width of CI: 0.02–0.406), test performance was rated poor to fair for DLR+ and good for DLR−, similar to the main analysis.

In the fresh vulvar smears, the kit was positive in 5.45% of dysplasia-free samples, rising to 77.78% in VHSIL (VIN III). In VSCCs, 75.00% of the samples were positive.

The sensitivity for the fresh vulvar smears was 94.12% and the specificity 90.91%. With a DLR+ of 10.353 and a DLR− of 0.065, the test performance is excellent.

Further test quality criteria can be found in Table 2.

**Table 2:** GynTect® test quality criteria of vulvar samples

Main analysis (VLSIL, VHSIL, and VSCC = diagnosis positive)				
	FFPE		Vulvar smears	
	%	95%-width of CI	%	95%-width of CI
Sens.	94.44%	87.51–98.17%	94.12%	71.31–99.85%
Spec.	40.00%	12.16–73.76%	90.91%	80.05–96.98%
PPV	93.41%	86.2–97.54%	76.19%	52.83–91.78%
NPV	44.44%	13.7–78.8%	98.04%	89.55–99.95%
Prev.	90.00%	82.4–95.1%	23.60%	14.4–35.1%
DLR+	1.574	0.947–2.617	10.353	4.451–24.081
DLR−	0.139	0.044–0.435	0.065	0.01–0.434
Additional analysis (VHSIL and VSCC = diagnosis positive)				
	FFPE			
	%	95%-with of CI		
Sens.	97.37%	90.82–99.68%		
Spec.	29.17%	12.62–51.09%		
PPV	81.32%	71.78–88.72%		
NPV	77.78%	39.99–97.19%		
Prev.	76.00%	66.40–85.00%		
DLR+	1.375	1.061–1.782		
DLR−	0.09	0.02–0.406		

### Impact of cervical dysplasia on the vulvar methylation

An incidental finding was that patients with cervical dysplasia had a higher proportion of GynTect® kit-positive vulvar smears than patients without cervical dysplasia. Therefore, we included dysplasia-free vulvar smears of 84 patients with cervical dysplasia and collected for 56 of them cervical smears as well. Of these, 13.10% (*n* = 11) had a CLSIL (CIN I), 29.76% (*n* = 25) a CHSIL (CIN II), 53.57% (*n* = 45) CHSIL (CIN III), and 3.57% (*n* = 3) a cervical carcinoma.

**Fig. 6:**
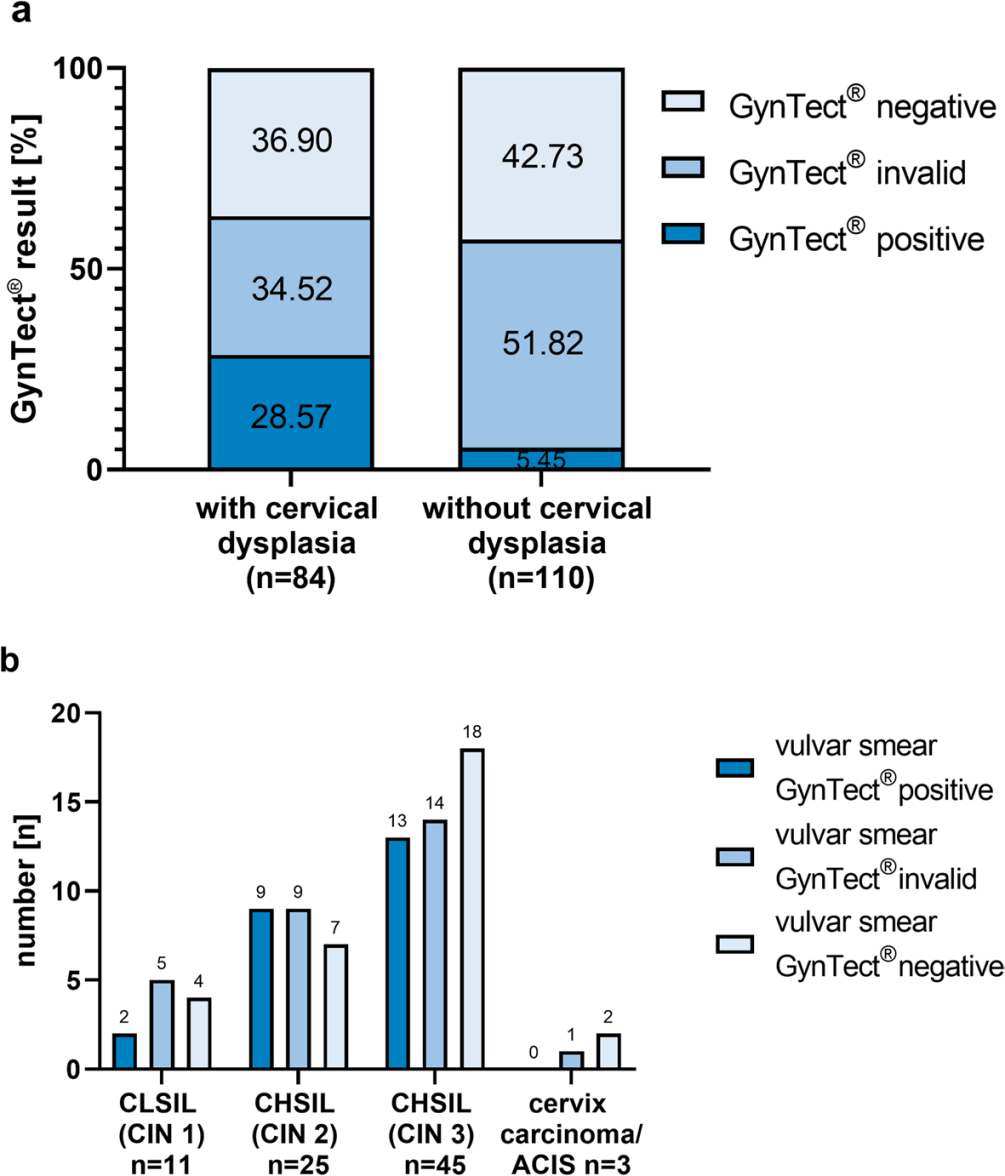
Comparison of dysplasia-free vulvar smear GynTect® results with and without cervical dysplasia (**a**). The variables differ significantly according chi-square test (*p* = 0). Distribution of the dysplasia-free vulvar smear GynTect® results from patients with different levels of cervical dysplasia (**b**). Number *n* on bars. ACIS, adenocarcinoma in situ of the cervix; CHSIL, cervical high-grade squamous intraepithelial lesion; CIN, cervical intraepithelial neoplasia; CLSIL, cervical low-grade squamous intraepithelial lesion

While patients without cervical dysplasia had 5.45% positivity in the methylation kit, those with concomitant cervical dysplasia showed positivity of 28.57% (Fig. 6a). The variables differ significantly according to the chi-square test (*p* = 0) (Additional file 2: Table S2). When the vulvar smear was positive in GynTect®, the corresponding cervical smear of that patient was always positive as well.

Figure 6b shows that the amount of GynTect®-positive dysplasia-free vulvar smears increases when the patient has a cervical dysplasia of higher grade. While two samples of patients with CLSIL (CIN I) appear positive in their dysplasia-free vulvar smear, it is nine patients with CHSIL (CIN II) and 13 patients with CHSIL (CIN III). The three patients with cervical carcinoma did not show a positive GynTect® result in the dysplasia-free vulva.

## Discussion

### Non-invasive clinical diagnosis of vulvar dysplasia using methylation markers

In this study, the use of DNA methylation markers for the detection of vulvar dysplasia was validated in a large cohort. The GynTect® methylation panel used achieved identical results in FFPE tissue as in fresh carcinoma tissue, thus the results are representative.

In addition, this study validated the use of DNA methylation markers on non-invasive vulvar smears. To our knowledge, we are the first to investigate this. Vulvar smears showed an even higher accuracy in detecting vulvar dysplasia than in FFPE tissue, probably due to the better material quality. The smears show a strong correlation between kit positivity and dysplasia level.

The best performance was achieved with the full GynTect® marker panel of six methylation markers, as already validated for cervical dysplasia [26]. The main analysis with all vulvar dysplasia and VSCC being classified as “diagnosis positive” showed good sensitivity and specificity. However, the test quality was according to the positive and negative diagnostic likelihood ratios fair to good, but not excellent [30]. An additional analysis of only high-grade dysplasia and VSCC being classified as “diagnosis positive” showed higher sensitivity and lower specificity than the main analysis. Five of the six markers (*ASTN1*, *ITGA4*, *RXFP3*, *SOX17*, and *ZNF671*) showed good positivity in vulvar dysplasia but with unexpected high positivity in VLSIL (VIN I) FFPE samples compared to higher dysplasia grades. If this result would be confirmed in vulvar smears, it would indicate a risk of overdiagnosis and overtreatment when using the GynTect® kit for these patients without adapting the thresholds and markers. Although showing a positive GynTect® result, the 11 VLSIL (VIN I) FFPE samples showed the lowest score with a mean of 10.36 (Additional file 3: Fig. S2). With a larger cohort, optimization of thresholds of the different markers to vulvar smears, and adapted marker scores, it is possible that we would not see that high positivity.

Interestingly, there is a high proportion of kit positivity in the dysplasia-free samples, but only in the FFPE material. This can be explained by the fact that the biopsies were always taken due to a macroscopic change. An inflammation or a change in the microenvironment and the methylation pattern in the biopsy tissue can therefore not be ruled out. This problem could not be avoided, as ethics prohibit the surgical and thus invasive removal of tissue samples from healthy patients. The vulvar smears, on the other hand, were largely generated from patients from the emergency room and hence represent the healthier collective. The very low positive rate of 5.45% (more on this below) thus supports the hypothesis.

Looking at the performance of the individual markers, the best-performing marker was *ASTN1*. In the kit, this has a score of 2 in the GynTect® test when used in cervical cancer diagnostics. Based on the findings, we suggest a higher score of *ASTN1* in a vulva diagnostic kit. We also recommend downgrading the marker *ZNF671*, which had the highest score of 6 in the cervix kit, to 2 points. *DLX1*, by far the worst-performing marker in vulvar tissue, is positive in 80% of dysplasia-free controls. It offers no additional diagnostic value and should therefore not be used in a vulvar methylation kit. A detailed analysis of the performance of all individual markers is provided in the supplements (Additional file 1).

The current state of relevant studies on methylation markers in vulvar dysplasia and VSCC is sparse. For VSCC and VSIL, somatic mutations have been investigated in several studies with targeted or whole exome sequencing [19–22, 34–37]. Unfortunately, for VSCC, the characterization of epigenetic changes has been attempted in only a few studies [24, 38–43]. For the most part, only one or two markers were examined. However, our results in FFPE tissue are consistent with those of Thuijs et al. They demonstrated a correlation between methylation levels and severity of vulvar diagnosis in HPV-associated lesions, but they analyzed other methylation markers [24].

The cut-off values, scores, and deltas in the methylation kit are optimized for the cervix and should be adapted for a diagnostic kit for vulvar tissue, including the scoring of the markers described above. This would most likely improve the DLR+/− rating, which would then be in line with the other well-performing test quality criteria. The high number of invalid samples may be explained by two facts: the strict evaluation criteria for GynTect® and the amount of cell material obtained from the smears. Significantly less sample material was obtained from vulvar smears than from cervical smears, as the smear material was primarily skin rather than mucous membrane. Due to less cell material, the Ct value of the control marker *IDS-M* in the qRT-PCR was correspondingly higher than the defined maximum value of 32 cycles. Accordingly, the results of the samples were evaluated as invalid. This range should be re-evaluated if the kit is used for vulvar smears in the future. Furthermore, the vulvar smear must be taken carefully to ensure that sufficient sample material is obtained. In our study, a cytobrush proved to be best suited for this purpose.

For this study, the positive samples in the kit were labeled positive if the kit was positive overall despite a single increased *IDS-M* value. Otherwise, the kit was considered invalid. Consequently, the calculations of sensitivity and specificity should still be interpreted with caution until a vulva-optimized evaluation has been performed, as the *IDS-M* limits could distort the evaluation.

Not all the VSCCs were detected with the methylation kit (VSCC 82.93%). However, some other promising markers are reported in the literature and could be included in a diagnostic kit [23, 24, 44, 45]. Yet, these markers have only been studied to a limited extent and require further research beforehand.

Encouragingly, dysplasia-free tissue is well detected as negative in the vulvar smears. The few dysplasia-free samples with a positive methylation kit (*n* = 6) were explored further by our research team. Despite a negative dysplasia status at the time of the vulvar smear, positive patients were still predominantly a high-risk group.

The data from the prevalent study shows no correlation between methylation status and hrHPV status. This deviates from our initial assumption. A study by Wakeham et al. from 2017 showed a similar trend, but less pronounced. There, the proportion of HPV-positive samples for VLSIL was 86% (vs. 76.92% here), for VHSIL 88% (vs. 66.68% here) and VSCCs 46.6% (vs. 9% here) [46]. No explanation was offered there due to a different study focus. We find evidence for this hypothesis in several places:

1. In samples with a positive methylation test, hrHPV status is almost equally positive and negative. The kit therefore also detects methylation markers in hrHPV-negative samples and tests. This indicates that methylation of the markers is independent of HPV infection. This finding may seem somewhat surprising seen in the light that these markers were identified in cervical cancer, which almost always is evoked by HPV infection. On the other hand, methylation of these markers has not yet been assessed in HPV-negative malignancies.
2. The proportion of hrHPV-positive samples decreases with increasing dysplasia grade. The reasons for this are most likely multifactorial: It is known that hrHPV-induced dysplasia shows a milder course than dVIN and is less likely to develop into a VSCC [9, 47]. Systematic hrHPV testing as part of cervical cancer screening enables patients to be diagnosed and treated at an early stage in the dysplasia unit. Patients with dVIN do not have this temporal advantage, so they are often first diagnosed with higher-grade dysplasia or even carcinoma. A bias can be assumed due to the earlier detection and consecutively earlier remediation of hrHPV-associated vulvar HSIL, so that the hrHPV-negative samples predominate with increasing dysplasia grade/VSCC. Furthermore, the data basis is limited due to the high proportion of invalid samples, particularly in the case of VSCC. In addition, hrHPV detection in vulvar lesions, in contrast to cervical dysplasia, is not standard—neither by testing nor by immunohistochemical staining. Moreover, the latter is only an indirect detection in the form of overexpression of cyclin-dependent kinase inhibitor 2A (p16^ink4a)^. The reproducibility of p16^ink4a^ interpretation in cytologic specimens is a concern, and the possible p16^ink4a^ positivity observed in non-dysplastic cells poses a problem in the evaluation of cytologic specimens [48, 49]. To date, there is no hrHPV test approved for vulvar tissue. The use of a cervical hrHPV test is hindered by the small amount of cell material in vulvar smears. HPV detection is therefore not standard and is further complicated both prospectively and retrospectively.
3. Our data shows that the methylation kit also works in dVIN, which are etiologically hrHPV-independent, as well as in hrHPV-negative VSIL. It is known that dVIN shows positive methylation rates. In the prevalent study, five out of the 11 dVIN samples tested positive (45.45%), two tested negative, and four tested invalid in the methylation panel. Thuijs et al. also examined dVIN samples. Like in the prevalent study, it was a small sample size (4 isolated dVIN and 12 dVIN adjacent to a VSCC). The markers showed higher methylation levels with increasing severity of disease in hrHPV negative samples. Unfortunately, dVIN without VSCC was only tested for 50% of the markers (6/12), due to limited DNA availability [24]. We would like to emphasize that dVIN showed positive methylation in the prevalent study, but no differences in incidence compared to hrHPV positive samples.

We advocate standard HPV testing for vulvar dysplasia and carcinoma, to eradicate the high proportion with unknown HPV status for more reliable results. For that it would need a specific study comparing the HPV status by p16^ink4a^ positivity and HPV PCRs with bigger sample size, to answer if a vulvar HPV test should be recommended and has clinical relevance, which was not the focus of our study now.

Limiting factors include the small sample size in some of the subgroups. Despite a substantial total sample size of 121 FFPE samples and 237 fresh vulvar cell smears, only small quantities of some samples could be generated. This might affect the robustness of the findings. For example, there were a total of 11 dVIN samples. This is consistent with problems in other studies [24]. The low number of dVIN in this group can be explained by the fact that most dVINs are recognized at time of VSCC diagnosis and not prior to VSCC diagnosis. Nine samples of VHSIL (VIN III) fresh vulvar swabs could be obtained despite intensive screening. An explanation for this could be the earlier detection and treatment by HPV testing and is consistent with the internal incidence statistics for the smear test period.

In addition, the panel has only been industrially validated for cervical tissue. For example, no specific thresholds have been defined for vulvar tissue. This could have a further impact on the results and will be an area for future work.

Furthermore, there was no follow-up of the patients, but this is planned for a follow-up study.

### Methyltransferases in vulvar dysplasia

Maintenance of DNA methylation by the DNMT is critical during development and in transcriptional regulation. However, aberrant expression of the DNMT has been reported for several human cancers, including those of epithelial origin and cervical dysplasia [50–52]. DNMT3a is primarily responsible for early de novo methylation, especially in embryonic development. DNMT1 is primarily responsible for the transfer of methylation patterns after cell division. Our data support this. The new and altered methylation happens early. With high-grade dysplasia, DNMT1 is expressed to pass on the methylation as the cells divide [53].

In our study, DNMT1 expression could only be detected in VHSIL (VIN III) and VSCC samples. This implies that the DNMT1 expression is upregulated only in advanced stages of vulvar carcinogenesis. DNMT3a expression could be detected in all dysplasia grades including dysplasia-free vulvar tissue. Its expression is significantly higher compared to the dysplasia-free tissue. The fold change in DNMT3a expression compared to dysplasia-free tissue is highest in VLSIL (VIN I) and then decreases as the dysplasia progresses. This shows that DNMT3a expression is strongly increased in the early phase of vulvar carcinogenesis, implying that changes in DNA methylation occur most frequently at the beginning of vulvar carcinogenesis. This corresponds with the methylation-kit results where the marker genes are already methylated early in vulvar carcinogenesis. The results also suggest that increased DNMT3a expression, and thus probably increased DNA methylation, occurs throughout the entire vulvar carcinogenesis.

To date, expression of the DNMT in VSCC has been examined in only one study by Leonard et al., who found overexpression of DNMT3A in the invasive component of vulvar tumors in 44% of samples. This was moreover associated with an increased risk of local vulvar recurrence. DNMT1 was over-expressed in 83% of tumors [38]. The overexpression is consistent with our results.

### The methylation kit should be used with caution in visible condylomatous skin changes and lichen sclerosus and planus.

The results reveal potential limitations for the application of the methylation kit on vulvar tissue.

According to 2015 ISSVD terminology, a synonym for VLSIL (VIN I) is flat condyloma. These condylomatous skin changes are associated with HPV low-risk [6]. Our results show a high percentage of positivity of 76.92% in the methylation kit for VLSIL (VIN I). It can be concluded from the results that the methylation kit should be used with caution and awareness in order to avoid a possible overdiagnosis of apparent condylomatous skin changes, as vulvar dysplasia may be falsely recognized.

A similar situation applies to lichen sclerosus and planus of the vulva. Samples with lichen sclerosus and planus changes with and without additional histologically confirmed dVIN were analyzed with the methylation kit. The proportion of positive samples in the kit did not differ between the groups. Plus, lichen without additional dysplasia are more frequently positive than the dysplasia-free smears without lichen (36.36% versus 5.45% positivity).

Recent studies of Voss et al. concluded in their work on methylation markers in vulvar tissue that methylation assays can be useful and prognostic biomarkers, particularly for HPV-independent precursor lesions that resemble reactive or inflammatory nondysplastic lesions [45] and patients with lichen sclerosus [54]. However, we were not able to repeat those results in our smaller patient cohort with a different marker panel and draw a more careful conclusion here: due to overlaps in methylation kit results, the assessed methylation markers should be applied with awareness in cases of vulvar lichen sclerosus or vulvar lichen planus to avoid false positive results.

### DNA methylation in genital dysplasia appears to be more extensive than previously thought

Despite normal findings on the vulva, patients with cervical dysplasia had a significantly higher proportion of positive kit results in vulvar tissue than patients without cervical dysplasia.

There is no explanation for this in the comparative literature. There are two possible explanations:

1. HPV infection is not as local as previously thought. As discussed above, DNA methylation in vulvar dysplasia appears to have no hrHPV association. This explanation therefore seems rather unlikely.
2. DNA methylation is not as local as previously assumed. Cervical dysplasia appears to have a measurable and detectable effect far beyond the cervical tissue. The concept of multicentric lower genital tract disease, defined as intraepithelial lesions or cancer at two or three sites (cervix, vagina, and vulva), is well recognized. Multiple primary preinvasive or invasive lesions of the cervix, vagina, vulva, perianal area, and anus can occur synchronously or metachronously [55–57]. So far, however, only local changes have been assumed [58]. However, at least the epigenetic level in the form of methylation patterns seems to have significantly more extensive effects throughout the genital tissue. In some cases, the tumor environment appears to extend from the cervix to the vulva.

This sheds new light on epigenetics and would have a direct impact on the diagnosis, monitoring, and treatment of the risk collective. Patients with (suspected) vulvar and cervical dysplasia should subsequently also be examined for other genital dysplasias. For example, vulvar, cervical, and vaginal colposcopy as well as HPV testing could be included in the diagnostic standard and follow-up care for patients with genital dysplasia or carcinoma.

## Conclusions

This study’s findings show that the diagnostic methylation kit by GynTect® is suitable for detecting vulvar neoplasia. Validation on vulvar smears demonstrated even higher accuracy than in FFPE tissue. However, adjustments may be necessary to achieve comparable specificity, and further research is needed to optimize the evaluation of vulvar smears and explore additional markers for inclusion in the diagnostic kit. Lichen sclerosus and planus should be considered when interpreting results, and the kit should be used with caution for patients with lichen sclerosus and planus. We found that methylation changes occur early, with the highest positivity in VLSIL (VIN I). Surprisingly, the findings suggest that DNA methylation patterns in vulvar tissue may not be as locally confined as previously presumed. This provides new insights into cancer epigenetics and highlights the need for further research to better understand the broader implications of methylation markers in non-invasive clinical diagnosis of vulvar dysplasia.

## Supplementary Information

Additional file 1: Table S1 Anonymous patient data and original values

Additional file 2: Table S2 Chi-square tests data of Figs. 1, 4, and 6a

Additional file 3: Figures S1–S3. Fig. S1 Comparison of GynTect® scores between fresh frozen carcinoma and the corresponding FFPE sample. FFPE, formalin-fixed paraffin-embedded*.* Fig. S2 Boxplots of GynTect® scores of all (**a**) FFPE or only GynTect®-positive FFPE samples (**b**). Median values, 95% interval ± SEM are shown. FFPE, formalin-fixed paraffin-embedded; VHSIL, vulvar high-grade squamous intraepithelial lesion; VIN, vulvar intraepithelial neoplasia; VLSIL, vulvar low-grade squamous intraepithelial lesion; VSCC, vulvar squamous cell carcinoma*.* Fig. S3 Boxplots of Ct values of all (**a**) FFPE or only GynTect®-positive FFPE samples (**b**). Median values, 95% interval ± SEM are shown. FFPE, formalin-fixed paraffin-embedded; VHSIL, vulvar high-grade squamous intraepithelial lesion; VIN, vulvar intraepithelial neoplasia; VLSIL, vulvar low-grade squamous intraepithelial lesion; VSCC, vulvar squamous cell carcinoma

## Data Availability

All data generated or analyzed during this study are included in this published article and its supplementary information files.

## Abbreviations

ACIS: Adenocarcinoma in situ of the cervix
°C: Degrees Celsius
CI: Confidence interval
CIN: Cervical intraepithelial neoplasia
CpG: Cytosine-phosphate-guanine
Ct: Cycle threshold
(c)DNA: (Complementary) deoxyribonucleic acid
DEPC: Diethyl pyrocarbonate
DLR+/−: Positive/negative diagnostic likelihood ratio
DNMT: DNA methyltransferase
FFPE: Formalin-fixed paraffin-embedded
g: Gram
h: Hour(s)
HPV: Human papilloma virus
hr: High risk
(C/V)HSIL: (Cervical/vulvar) high-grade squamous intraepithelial lesion
ISSVD: International Society for the Study of Vulvovaginal Disease
l: Liter
(C/V)LSIL: (Cervical/vulvar) low-grade squamous intraepithelial lesion
m: Meter
M: Molar
min: Minute(s)
NPV: Negative predictive value
(qRT-)PCR: (Quantitative real-time) polymerase chain reaction
pH: Potential of hydrogen
PPV: Positive predictive value
Prev.: Prevalence
rpm: Revolution per minute
s: Second(s)
Sens.: Sensitivity
Spec.: Specificity
SST: Somatostatin
VAIN: Vaginal intraepithelial neoplasia
(d/u)VIN: (Differentiated/usual) vulvar intraepithelial neoplasia
VSCC: Vulvar squamous cell carcinoma
×g: Relative centrifugal force
