# Supplementary material for: Non-invasive diagnosis of vulvar dysplasia using cervical methylation markers—a case control study
Source: BMC Med. 2025 Feb 28;23:128. doi: 10.1186/s12916-025-03954-x (PMC11871814; doi:10.1186/s12916-025-03954-x)
Supplement: Supplementary file 3 — Additional file 3: Figures S1–S3. Fig. S1 Comparison of GynTect® scores between fresh frozen carcinoma and the corresponding FFPE sample. FFPE, formalin-fixed paraffin-embedded. Fig. S2 Boxplots of GynTect® scores of all (a) FFPE or only GynTect®-positive FFPE samples (b). Median values, 95% interval ± SEM are shown. FFPE, formalin-fixed paraffin-embedded; VHSIL, vulvar high-grade squamous intraepithelial lesion; VIN, vulvar intraepithelial neoplasia; VLSIL, vulvar low-grade squamous intraepithelial lesion; VSCC, vulvar squamous cell carcinoma. Fig. S3 Boxplots of Ct values of all (a) FFPE or only GynTect®-positive FFPE samples (b). Median values, 95% interval ± SEM are shown. FFPE, formalin-fixed paraffin-embedded; VHSIL, vulvar high-grade squamous intraepithelial lesion; VIN, vulvar intraepithelial neoplasia; VLSIL, vulvar low-grade squamous intraepithelial lesion; VSCC, vulvar squamous cell carcinoma [file 12916_2025_3954_MOESM3_ESM.docx]

**Supplements**

### Scores in GynTect^®^ methylation kit

The methylation kit achieved an overall average score of 6.94 for FFPE tissue. When looking at the scores for each respective kit result, the kit-positive samples achieved an average score of 11.74. The negative samples had an average score of 3.2, and the invalid samples an average score of 5.9.

## Performance of the individual methylation markers

### Astrotactin-1 (*ASTN1*)

The marker Astrotactin-1 (*ASTN1*) is scored 2 out of 15 in the methylation kit for cervix in case of positivity.

For *ASTN1*, an abrupt increase from controls to dysplasia tissue can be seen in the bar chart for FFPE tissue. From 50.00% kit-positive samples in dysplasia-free tissue, there is an increase to 100.00% positive samples for VIN I and VIN II, 94.12% in VIN III and 100.00% in carcinoma. There is no gradual increase across the dysplasia stages. An analysis of the test quality criteria shows a sensitivity of 98.1% and a specificity of 46.67% for *ASTN1* in the FFPE samples. A DLR+ of 1.839 and DLR- of 0.041 result in a fair to good test performance.

For the fresh vulvar smears, the positivity increases gradually across the dysplasia stages from 12.61% for dysplasia-free tissue to 77.87% for VIN III and finally 100.00% for carcinoma. A sensitivity of 82.35% and specificity of 83.33% could be achieved (see Table S1). The DLR+ increased to 4.941, the DLR- was 0.212, showing a fair to good test performance for the fresh vulvar smears.

Table S1: Performance of ASTN1

| *ASTN1* | FFPE | | Vulvar smears | |
| --- | --- | --- | --- | --- |
|  | % | 95%-width of CI | % | 95%-width of CI |
| Sens: | 98.1% | 93.29% - 99.77% | 82.35% | 56.57% - 96.20% |
| Spec: | 46.67% | 21.27% - 73.41% | 83.33% | 75.20% - 89.66% |
| PPV: | 92.79% | 86.29% - 96.84% | 42.42% | 25.48% - 60.78% |
| NPV: | 77.78% | 39.99% - 97.19% | 96.94% | 91.31% – 99.36% |
| Prev: | 87.5% | 80.2% - 92.8% | 13.00% | 7.70% - 20.00% |
| DLR+ | 1.839 | 1.145 - 2.955 | 4.941 | 3.101 – 7.872 |
| DLR- | 0.041 | 0.009 - 0.178 | 0.212 | 0.076 – 0.593 |

### Distal-Less Homeobox 1 (*DLX1*)

Distal-Less Homeobox 1 (*DLX1*) is scored 1 out of 15 in the methylation kit for cervix in case of positivity.

*DLX1* is positive in all the samples for FFPE-tissue in every dysplasia stage. Discrimination of the dysplasia stages is not possible. Accordingly, the test quality criteria showed a sensitivity of 100.00% (95%-width of CI: 96.55% - 100.0%) and a specificity of 0.0% (95%-width of CI: 0.0% - 20.59%). DLR+ is 1.0, DLR- could not be calculated. This results in an overall fair to poor test performance for *DLX1* for FFPE tissue.

For the fresh vulvar smears, the positivity is also high for all stages, with 80.18 % for dysplasia-free tissue, 77.78% for VIN III and 100.00% for carcinoma. The fresh vulvar smears showed a sensitivity of 90.48% and a specificity of 16.04%. The DLR+ in the smears was 1.078, and the DLR- was 0.594, which corresponds to a fair to poor test performance (see Table S2).

Table S2: Performance of DLX1

| *DLX1* | FFPE | | Vulvar smears | |
| --- | --- | --- | --- | --- |
|  | % | 95 %-width of CI | % | 95%-width of CI |
| Sens: | 100.0% | 96.55% - 100,0% | 90.48% | 69.62% - 98.83% |
| Spec: | 0.0% | 0.0% - 20.59% | 16.04% | 9.63% - 24.43% |
| PPV: | 86.78% | 79.42% - 92.25% | 17.59% | 10.94% - 26.10% |
| NPV: | - | - | 89.47% | 66.86% - 98.70% |
| Prev: | 86.8% | 79.4% - 92.2% | 16.50% | 10.50% - 24.20% |
| DLR+ | 1.0 | 1.0 - 1.0 | 1.078 | 0.917 – 1.267 |
| DLR- | - | - | 0.594 | 0.148 – 2.380 |

### Integrin Subunit Alpha 4 (*ITGA4*)

Integrin Subunit Alpha 4 (*ITGA4*) is scored 2 out of 15 in the methylation kit for cervix in case of positivity.

We see an increase in *ITGA4*-positive FFPE samples, particularly towards carcinoma. Prior to this, the proportion of positive samples in the biological groups remains almost constant: 18.75% positive samples in dysplasia-free tissue, 15.38% in VIN I, 23.53% in VIN II, 23.53% in VIN III and the increase to 51.22% in vulvar carcinoma. The FFPE tissue showed a sensitivity of 33.02% (95%-width of CI: 24.19% - 42.82%) and a specificity of 78.57% (95%-width of CI: 49.2% - 95.34%), representing a fair to poor test performance.

For fresh vulvar smears, there is a gradual increase in positive samples across the biological groups. While 22.62% of controls are positive, the proportion increases to 44.44% for VIN III and 75.00% for carcinoma. In the fresh vulvar smears, there was a sensitivity of 61.90% and a specificity of 78.18% for *ITGA4* (see Table S3). The DLR+ was 2.837, the DLR- was 0.487, which is equivalent to a fair to poor test performance.

Table S3: Performance of ITGA4

| *ITGA4* | FFPE | | Vulvar smears | |
| --- | --- | --- | --- | --- |
|  | % | 95%-width of CI | % | 95%-width of CI |
| Sens: | 33.02% | 24.19% - 42.82% | 61.90% | 38.44% - 81.89% |
| Spec: | 78.57% | 49.2% - 95.34% | 78.18% | 69.30% - 85.49% |
| PPV: | 92.11% | 78.62% - 98.34% | 35.14% | 20.21% - 52.54% |
| NPV: | 13.41% | 6.89% - 22.74% | 91.49% | 83.92% - 96.25% |
| Prev: | 88.3% | 81.2% - 93.5% | 16.00% | 10.20% - 23.50% |
| DLR+ | 1.541 | 0.545 - 4.355 | 2.837 | 1.742 – 4.620 |
| DLR- | 0.852 | 0.629 - 1.156 | 0.487 | 0.280 – 0.848 |

### Relaxin Family Peptide Receptor 3 (*RXFP3*)

Relaxin Family Peptide Receptor 3 (*RXFP3*) is scored 2 out of 15 in the methylation kit for cervix in case of positivity.

Discrimination by biological group is possible in the bar chart for both FFPE and fresh vulvar smears. For FFPE tissue, there is a continuous increase from dysplasia-free tissue (18.75% positive) through the dysplasia stages (30.77% VIN I, 52.94% VIN II, 55.88% VIN III) to carcinoma (90.24% positive). This results in a sensitivity of 65.09% (95 %-width of CI: 55.22% - 74.1%) and a specificity of 80.0% (95%-width of CI: 51.91% - 95.67%). The DLR+ was 3.255, the DLR- 0.436, which corresponds to a fair to poor test performance (see Table S4).

The progression in kit positivity was also reflected in the fresh vulvar smears: 23.42% in the controls, 66.67% in VIN III to 75.00% in the carcinomas. In the fresh vulvar smears there was a sensitivity of 71.43% and a specificity of 76.15%. The DLR+ in the smears was 2.995, the DLR- was 0.375 which corresponds to a fair to poor test performance (see Table S4).

Table S4: Performance of RXFP3

| *RXFP3* | FFPE | | Vulvar smears | |
| --- | --- | --- | --- | --- |
|  | % | 95%-width of CI | % | 95%-width of CI |
| Sens: | 65.09% | 55.22% - 74.1% | 71.43% | 47.82% - 88.72% |
| Spec: | 80.0% | 51.91% - 95.67% | 76.15% | 67.03% - 83.79% |
| PPV: | 95.83% | 88.3% - 99.13% | 36.59% | 22.12% - 53.06% |
| NPV: | 24.49% | 13.34% - 38.87% | 93.26% | 85.90% - 97.49% |
| Prev: | 87.6% | 80.4% - 92.9% | 16.20% | 10.30% - 23.60% |
| DLR+ | 3.255 | 1.172 - 9.041 | 2.995 | 1.946 – 4.607 |
| DLR- | 0.436 | 0.304 - 0.627 | 0.375 | 0.189 – 0.744 |

### SRY-Box Transcription Factor 17 (*SOX17*)

SRY-Box Transcription Factor 17 (*SOX17*) is scored 2 out of 15 in the methylation kit for cervix in case of positivity.

The methylation marker *SOX17* showed a consistent increase in the proportion of positive samples across the dysplasia stages both for FFPE and in the smears. In FFPE tissue, positivity increased from 25.00% in controls to 46.15% in VIN I, 58.82% in VIN II, 70.59% in VIN III and 80.49% in carcinomas. *SOX17* had a sensitivity of 68.87% (95%-width of CI: 59.14% - 77.51%) and a specificity of 73.33% (95%-width of CI: 44.9% - 92.21%) for the FFPE samples. DLR + was 2.583, DLR- was 0.452, which corresponds to a fair to poor test performance (see Table S5).

The fresh vulvar smears showed an increase from 25.23% in the controls, 55.56% in VIN III to 83.33% in the carcinomas. Sensitivity and specificity were both similar to the FFPE tissue with 68.18% sensitivity and a specificity of 74.31%. The DLR+ was 2.654, the DLR- was 0.428, which corresponds to a fair to poor test performance (see Table S5).

Table S5: Performance of SOX17

| *SOX17* | FFPE | | Vulvar smears | |
| --- | --- | --- | --- | --- |
|  | % | 95%-width of CI | % | 95%-width of CI |
| Sens: | 68.87% | 59.14% - 77.51% | 68.18% | 45.13% - 86.14% |
| Spec: | 73.33% | 44.9% - 92.21% | 74.31% | 65.06% - 82.20% |
| PPV: | 94.81% | 87.23% - 98.57% | 34.88% | 21.01% - 50.93% |
| NPV: | 25.0% | 13.19% - 40.34% | 92.05% | 84.30% - 96.74% |
| Prev: | 87.6% | 80.4% - 92.9% | 16.80% | 10.80% - 24.30% |
| DLR+ | 2.583 | 1.105 - 6.036 | 2.654 | 1.730 – 4.073 |
| DLR- | 0.425 | 0.28 - 0.644 | 0.428 | 0.230 – 0.797 |

### Zinc finger protein 671 (*ZNF671*)

The most potent methylation marker in the detection of cervical dysplasia, Zinc finger protein 671 (*ZNF671*), is scored 6 out of 15 in the methylation kit for cervix in case of positivity.

Looking at the bar chart, FFPE tissue showed 50.00% positivity in the controls. 76.92% of VIN I were positive, 52.94% of VIN II and 61.76% of VIN III. In the carcinoma samples, 82.93% were positive. This resulted in a sensitivity of 70.48% (95%-width of CI: 60.78% - 78.98%) and a specificity of 50.00% (95%-width of CI: 24.65% - 75.35%). DLR+ was 1.41, DLR- 0.59, which equates to a fair to poor test performance (see Table S6).

Similar to the previous markers, there was a continuous increase in *ZNF671*-positive samples in the fresh vulvar smears across the biological groups: 24.32% positive samples in dysplasia-free tissue, 33.33% in VIN III and 91.67% in vulvar carcinoma. This resulted in a sensitivity of 63.64% and a specificity of 74.77%. DLR+ was 2.522 for the smears, DLR- was 0.486. This correlates to a fair to poor test performance (see Table S6).

Table S6: Performance of ZNF671

| *ZNF671* | FFPE | | Vulvar smears | |
| --- | --- | --- | --- | --- |
|  | % | 95%-width of CI | % | 95%-width of CI |
| Sens: | 70.48% | 60.78% - 78.98% | 63.64% | 40.66% - 82.80% |
| Spec: | 50.0% | 24.65% - 75.35% | 74.77% | 65.45% - 82.67% |
| PPV: | 90.24% | 81.68% - 95.69% | 34.15% | 20.08% - 50.59% |
| NPV: | 20.51% | 9.3% - 36.46% | 90.91% | 82.87% - 95.99% |
| Prev: | 86.8% | 79.4% - 92.2% | 17.10% | 11.00% - 24.70% |
| DLR+ | 1.41 | 0.85 - 2.336 | 2.522 | 1.602 – 3.971 |
| DLR- | 0.59 | 0.333 - 1.046 | 0.486 | 0.227 – 0.855 |

### Fresh frozen carcinomas


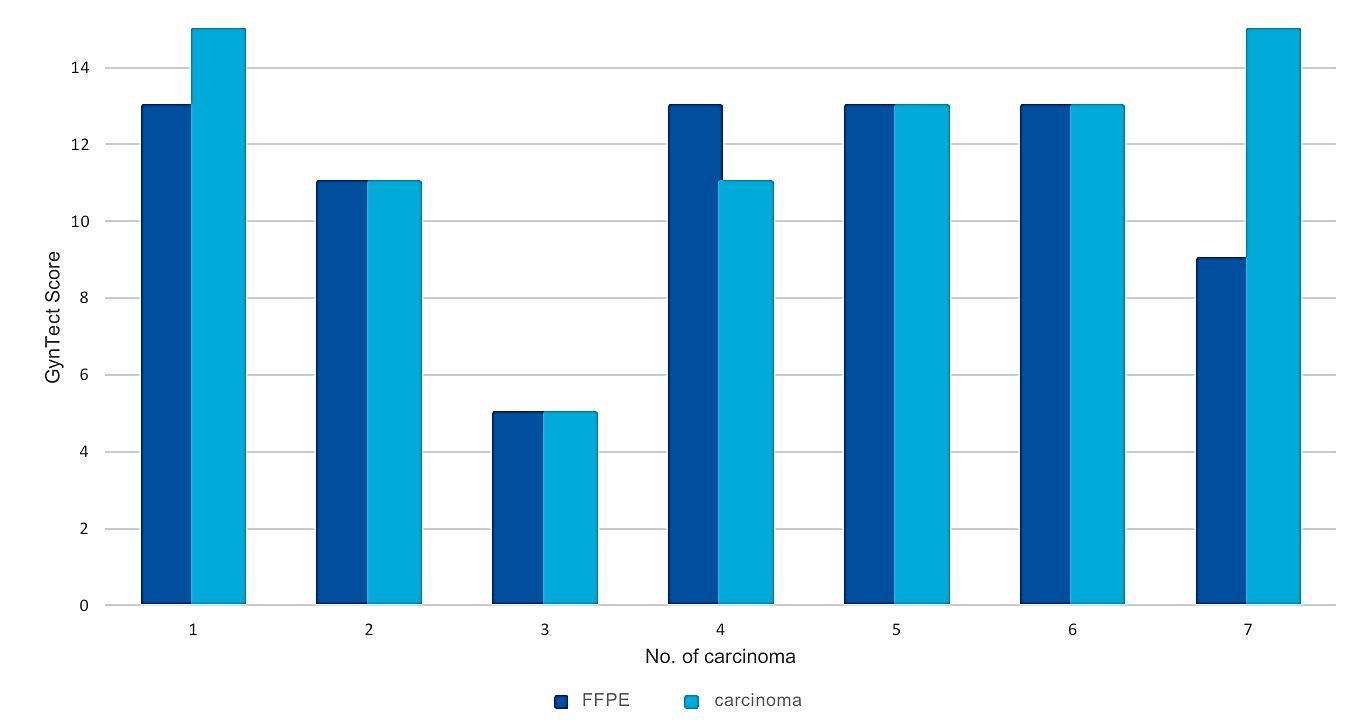


Figure S1: comparison of GynTect^®^ scores between fresh frozen carcinoma and the corresponding FFPE sample. FFPE, formalin-fixed paraffin-embedded


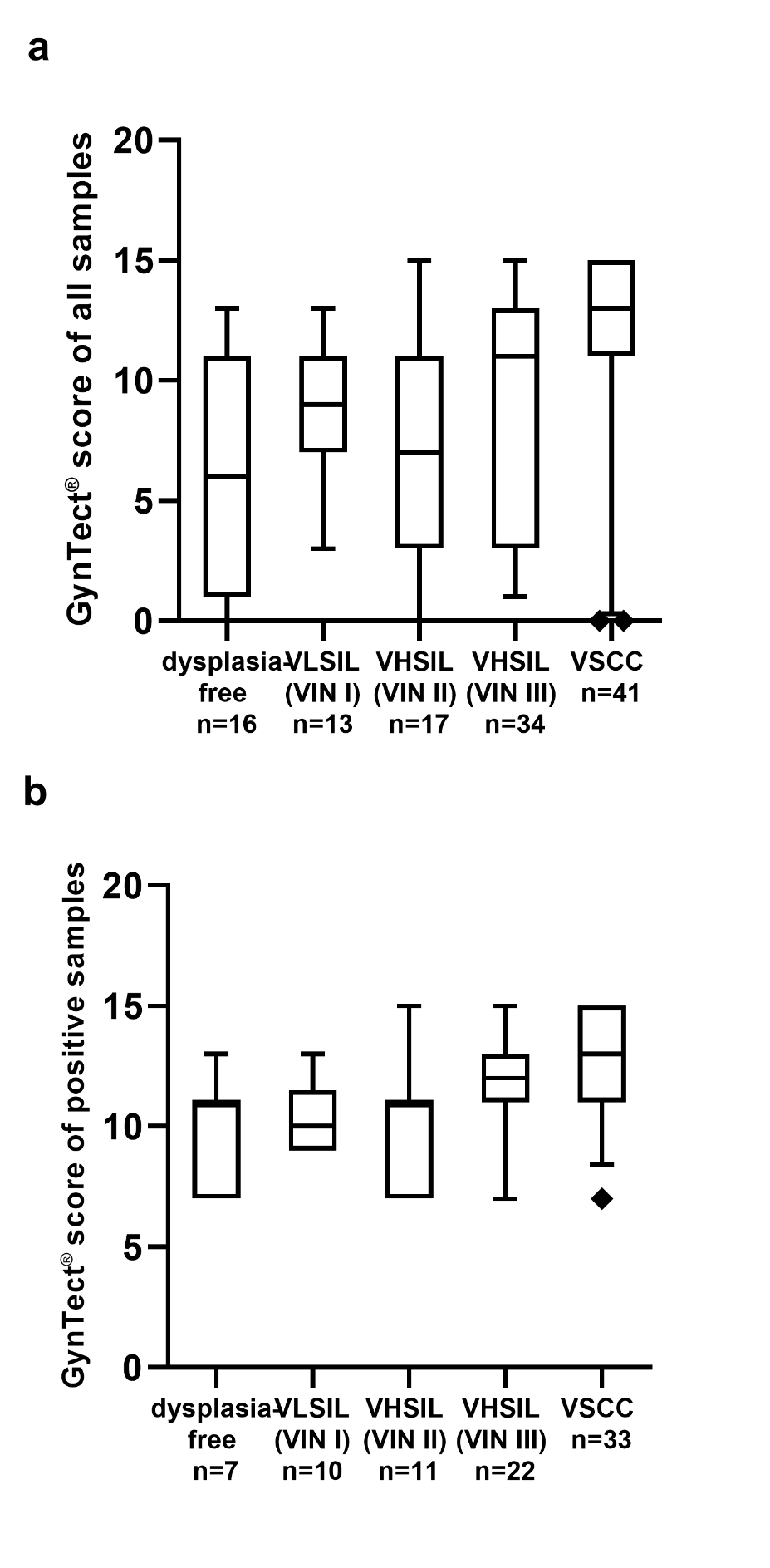


Figure S2: Boxplots of GynTect^®^ scores of all (**a**) FFPE or only GynTect^®^-positive FFPE samples (**b**). Median values, 95% interval ± SEM are shown. FFPE, formalin-fixed paraffin-embedded; VHSIL, vulvar high-grade squamous intraepithelial lesion; VIN, vulvar intraepithelial neoplasia; VLSIL, vulvar low-grade squamous intraepithelial lesion; VSCC, vulvar squamous cell carcinoma


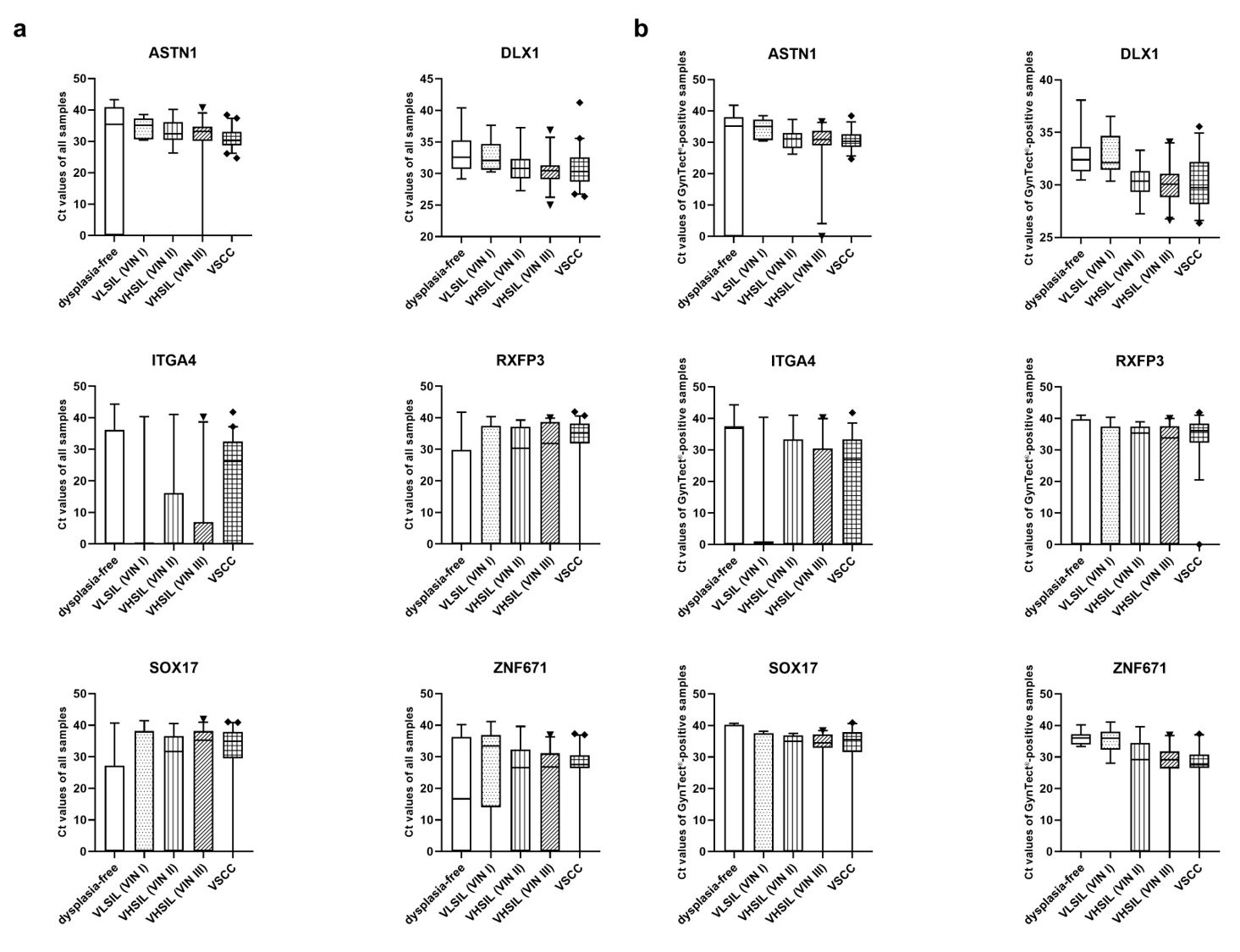


Figure S3: Boxplots of Ct values of all (**a**) FFPE or only GynTect^®^-positive FFPE samples (**b**). Median values, 95% interval ± SEM are shown. FFPE, formalin-fixed paraffin-embedded; VHSIL, vulvar high-grade squamous intraepithelial lesion; VIN, vulvar intraepithelial neoplasia; VLSIL, vulvar low-grade squamous intraepithelial lesion; VSCC, vulvar squamous cell carcinoma
